# Publication Delay of Randomized Trials on 2009 Influenza A (H1N1) Vaccination

**DOI:** 10.1371/journal.pone.0028346

**Published:** 2011-12-02

**Authors:** John P. A. Ioannidis, Lamberto Manzoli, Corrado De Vito, Maddalena D'Addario, Paolo Villari

**Affiliations:** 1 Stanford Prevention Research Center, Department of Medicine and Department of Health Research and Policy, Stanford University School of Medicine, Stanford, California, United States of America; 2 Section of Hygiene, Epidemiology, Pharmacology and Legal Medicine, University “G. d'Annunzio” of Chieti, Chieti, Italy; 3 Department of Public Health and Infectious Diseases, Sapienza University of Rome, Rome, Italy; University of Illinois-Chicago, United States of America

## Abstract

**Background:**

Randomized evidence for vaccine immunogenicity and safety is urgently needed in the setting of pandemics with new emerging infectious agents. We carried out an observational survey to evaluate how many randomized controlled trials testing 2009 H1N1 vaccines were published among those registered, and what was the time lag from their start to publication and from their completion to publication.

**Methods:**

PubMed, EMBASE and 9 clinical trial registries were searched for eligible randomized controlled trials. The units of the analysis were single randomized trials on any individual receiving influenza vaccines in any setting.

**Results:**

73 eligible trials were identified that had been registered in 2009–2010. By June 30, 2011 only 21 (29%) of these trials had been published, representing 38% of the randomized sample size (19905 of 52765). Trials starting later were published less rapidly (hazard ratio 0.42 per month; 95% Confidence Interval: 0.27 to 0.64; p<0.001). Similarly, trials completed later were published less rapidly (hazard ratio 0.43 per month; 95% CI: 0.27 to 0.67; p<0.001). Randomized controlled trials were completed promptly (median, 5 months from start to completion), but only a minority were subsequently published.

**Conclusions:**

Most registered randomized trials on vaccines for the H1N1 pandemic are not published in the peer-reviewed literature.

## Introduction

Randomized controlled trials are pivotal in providing reliable information about the effectiveness and safety of vaccines. In the case of rapidly emerging pandemics with newly discovered infectious agents, such as the 2009 influenza A(H1N1) virus, the availability of such information becomes even more time-sensitive [1]. While some preliminary information from such trials can be provided in confidential communications to regulatory and public policy authorities for immediate decisions, the scientific peer-review process offered by journals provides the ultimate possible guarantee about the quality of these data and the balanced presentation of the results. In an evolving, emerging pandemic for which a new vaccine is needed, it is usually possible to recruit a sufficient number of interested participants in limited time. Moreover, outcomes can be assessed quickly in vaccine trials when the primary emphasis is on immunological response (assessed in a few weeks) and short-term adverse events. However, are such trials published also quickly in the peer-reviewed literature?

To address this question, we evaluated empirically the publication delay of randomized trials of 2009 H1N1 vaccines [2]. We considered all trials of these vaccines registered in main trial registries in 2009 and 2010 and evaluated whether these trials have published any data in the peer-reviewed literature by the end of June 2011 and also how long it took from the time they started until they published their results.

## Methods

Randomized controlled trials evaluating 2009 influenza A(H1N1) vaccine immunogenicity and safety in healthy humans who had not previously received 2009 H1N1 vaccines were retrieved through searches in MEDLINE and EMBASE. We focused on trials that had been registered in at least one of several clinical trial registries (Cochrane Controlled Clinical Trial Register, ISRCTN, US ClinicalTrials.gov, WHO ICTRP, GSK Clinical Study Register, and Indian, Australian New Zealand and Chinese Clinical Trial Registries) in 2009 or 2010. We had no language restriction and the last update of searches for identifying published trials was performed on June 30, 2011. Search terms were “vaccine OR vaccines OR vaccination”, and “H1N1 OR pandemic” in all fields. The bibliographies of all relevant articles including reviews were reviewed for further references [2].

Randomized controlled trials were eligible for consideration regardless of the doses and formulations of the vaccine that they compared; the number of arms; the sample size; and whether they had been published or not. We screened potentially eligible registered trials to avoid double-entry of the same trial that may have been identified from two different sources. Moreover, whenever a trial had two or more publications of its results on the same sample size, we focused on the earliest published report in a peer-reviewed journal that provided any evidence on immunogenicity and/or safety in the study population. Whenever the same study published two or more reports with increasing/expanding sample sizes over time, we considered the incremental amount of evidence that became available at each publication, e.g. if a trial reported on 2000 patients in October 2009 and on 12000 patients in December 2009, we considered that randomized evidence on 2000 patients became available in the published literature in October 2009 and then evidence on another 10000 patients became available in December 2009.

For each eligible trial that had started and had been registered as starting before the end of 2010, we recorded the registry number; the sample size (actual, if completed; and anticipated, if not fully recruited yet); the sponsor(s); the date of starting; whether it was published or not; and the date of publication in the peer-reviewed literature for those trials that were published. For trials published online ahead of print, we used the time of electronic publication. We also collected information on the reported date of primary completion for trials that had been completed. Information on the date of completion may be less standardized across trials and thus less reliable, because occasionally some trialists and sponsors continue to report a trial as not yet completed even after it has published its main results, if there are plans for additional analyses or longer follow-up. Therefore, whenever the reported date of completion of a trial was within less than 3 months of its publication date (7 trials), we imputed the date of completion to be 3 months before the publication date. Unpublished trials with anticipated completion dates after June 30, 2011, are considered non-completed and time is censored on June 30, 2011 for all analyses.

We evaluated the time from starting a trial to its publication using Kaplan-Meier analysis considering all registered trials. We also evaluated with the log-rank test whether the time-to-publication was different for different sponsors, and then tested with Cox proportional hazards analysis whether there was any evidence that the risk of publication was dependent on the sponsor, sample size (log-transformed) and date of starting. We performed both univariate and multivariate analyses, in which we included a priori the three covariates above. Secondary analyses evaluated the time from starting a trial until its completion and the time from completion of a trial to its publication. The proportional hazards assumption was checked for all models using the Schoenfeld test and plotting Nelson-Aalen cumulative hazards estimates.

Finally, we evaluated using Spearman's rank correlation coefficient whether trials published early were selected for publication by journals with higher impact factor (according to Thomson ISI Journal Citation Reports, Edition 2009) than trials published later. Analyses were conducted in Stata 10.1 (Stata Corp., College Station, TX, USA, 2007). P-values are two-tailed.

## Results

**Figure 1:**
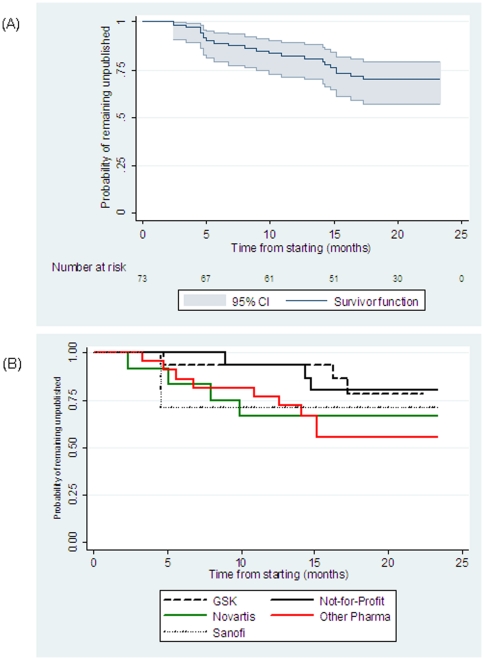
Time from start to publication for 2009 H1N1 vaccine trials overall (A) and according to sponsor (B).

We identified 73 randomized controlled trials of 2009 H1N1 vaccines that had been registered in 2009–2010. Of those, only 21 (29%) had been published by June 30, 2011. Figure 1A shows the Kaplan-Meier plot for the time-to-publication. The risk for a trial remaining unpublished was 69% at a year and a half after starting. The majority of the trials (57/73) had been sponsored by the industry testing vaccines manufactured by a total of 14 different companies (GSK n = 16, Novartis n = 12, Sanofi-Aventis n = 7, CSL n = 4, Panacea Biotec n = 4, Sinovac n = 2, Bharat Biotech n = 2, MedImmune n = 2, Baxter n = 2, Adimmune n = 2, Hualan Biological Bacterin n = 1, Novavax n = 1, VaxInnate Corporation n = 1, Vaxine Pty n = 1). Another 16 trials were sponsored by not-for-profit organizations, but each of these trials also tested vaccines from a single company with only three exceptions (NCT01000584, ISRCTN92328241, NCT00980850) that tested vaccines by two different companies. Our analysis showed no significant difference in the time-to-publication across the major sponsors (log-rank p-value = 0.39, figure 1B).

**Figure 2:**
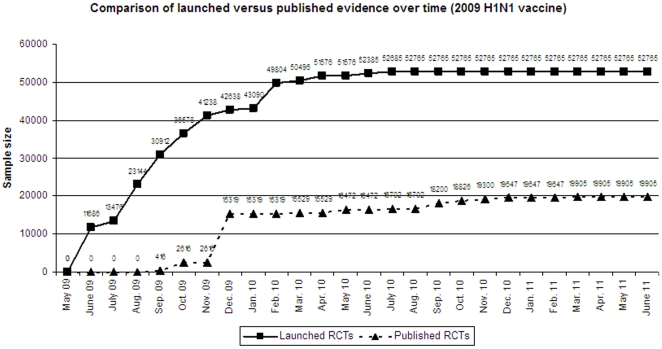
Cumulative sample size in launched and published trials of 2009 H1N1 vaccines over time.

Overall, the total sample size of the 73 trials amounts to 52765 participants. Of those, the 21 published trials include data on 19905 participants (38%). Figure 2 shows the total cumulative sample size over time of trials that were launched and of those that had been published over time. As shown, by November 2009, trials had been launched that cumulatively cover about 78% of the total randomized trial effort. Most of the remaining randomized evidence (total of 94%) had been launched by February 2010, and very little additional randomized evidence was collected in trials launched later in 2010. The published randomized data first appeared on September 10, 2009 with two small randomized trials published online in the New England Journal of Medicine (total n = 416), and the published evidence increased to n = 15319 by the end of the calendar year 2009, with a total of 9 trials published on 5 different vaccines. No other trials were published until March 2010, when the 2009–2010 pandemic season was ending in the Northern hemisphere. During the following 15 months, another 12 trials were published, all of them with modest sample sizes (107–1313 participants each).

In univariate analyses (Table 1), trials starting later were published far less rapidly (hazard ratio 0.42, 95% confidence interval (CI), 0.27 to 0.64 per month, p<0.001). In fact, none of the trials that started after October 2009 have been published as of June 30, 2011. The analysis found no trend for faster publication of larger trials (hazard ratio 0.93, 95% CI, 0.58 to 1.48, per 10-fold increase in sample size, p = 0.8). We also found no difference in the time-to-publication for trials sponsored by not-for-profit structures vs companies (hazard ratio 0.52, 95% CI, 0.15 to 1.78 per month, p = 0.3). Multivariate analyses confirmed univariate results: adjusting for sample size and sponsor (not-for profit vs companies), the hazard ratio of publication was 0.36 (95% CI, 0.23 to 0.58, p<0.001) for each month of later start.

**Table 1:** Predictors of time to completion and time to publication: hazard ratio (HR) and 95% confidence interval (CI) in univariate Cox models.

	Start to publication	Start to completion	Completion to publication
	HR (95% CI)	HR (95% CI)	HR (95% CI)
Calendar time (per 1 month later)	0.42 (0.27–0.64)	0.92 (0.85–1.02)	0.43 (0.27–0.67)
Sample size (per 10-fold increase)	0.93 (0.58–1.48)	0.98 (0.76–1.26)	0.88 (0.52–1.47)
Not-for-profit vs companies	0.52 (0.15–1.78)	0.99 (0.54–1.79)	0.48 (0.14–1.73)

**Figure 3:**
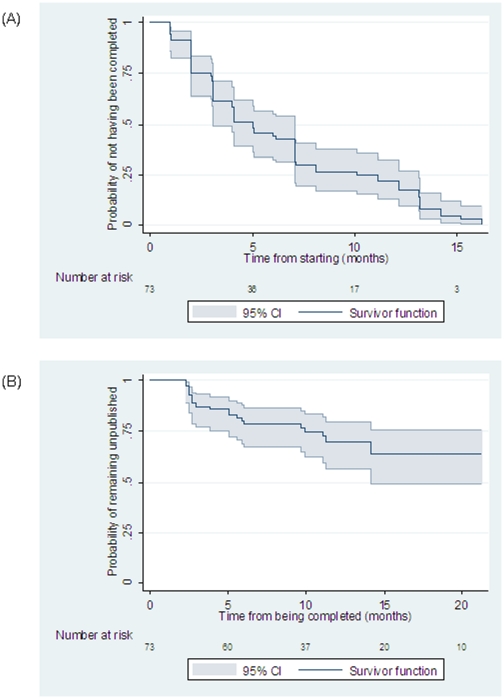
Time from start to completion (A) and from completion to publication (B) for 2009 H1N1 vaccine trials.

Figure 3 shows the Kaplan-Meier plot for the time from starting to completion (Figure 3A) and for the time from completion to publication of a trial (Figure 3B). The median time from starting to completion based on the Kaplan-Meier analysis was 5 months. We found that the time to completion did not differ for trials starting later, for those with company sponsors, or for those that were larger compared with earlier, not-for-profit, and smaller trials, respectively (Table 1).

In addition to the 21 published trials, 47 of the 52 unpublished RCTs were reported as completed (90.4%). After completion, some trials were published very fast (within 5 or less months), but then the publication rate declined; at 18 months after completion the estimated risk of remaining unpublished was 64%. Again, trials completed later were published less rapidly (hazard ratio 0.43, 95% CI, 0.27 to 0.67 per month p<0.001). Only one of the trials that were completed after April 2010 has been published as of June 30, 2011. The analysis showed no difference in the time-to-publication after completion in trials with different sponsors and sample sizes (both p>0.05) (Table 1).

**Figure 4:**
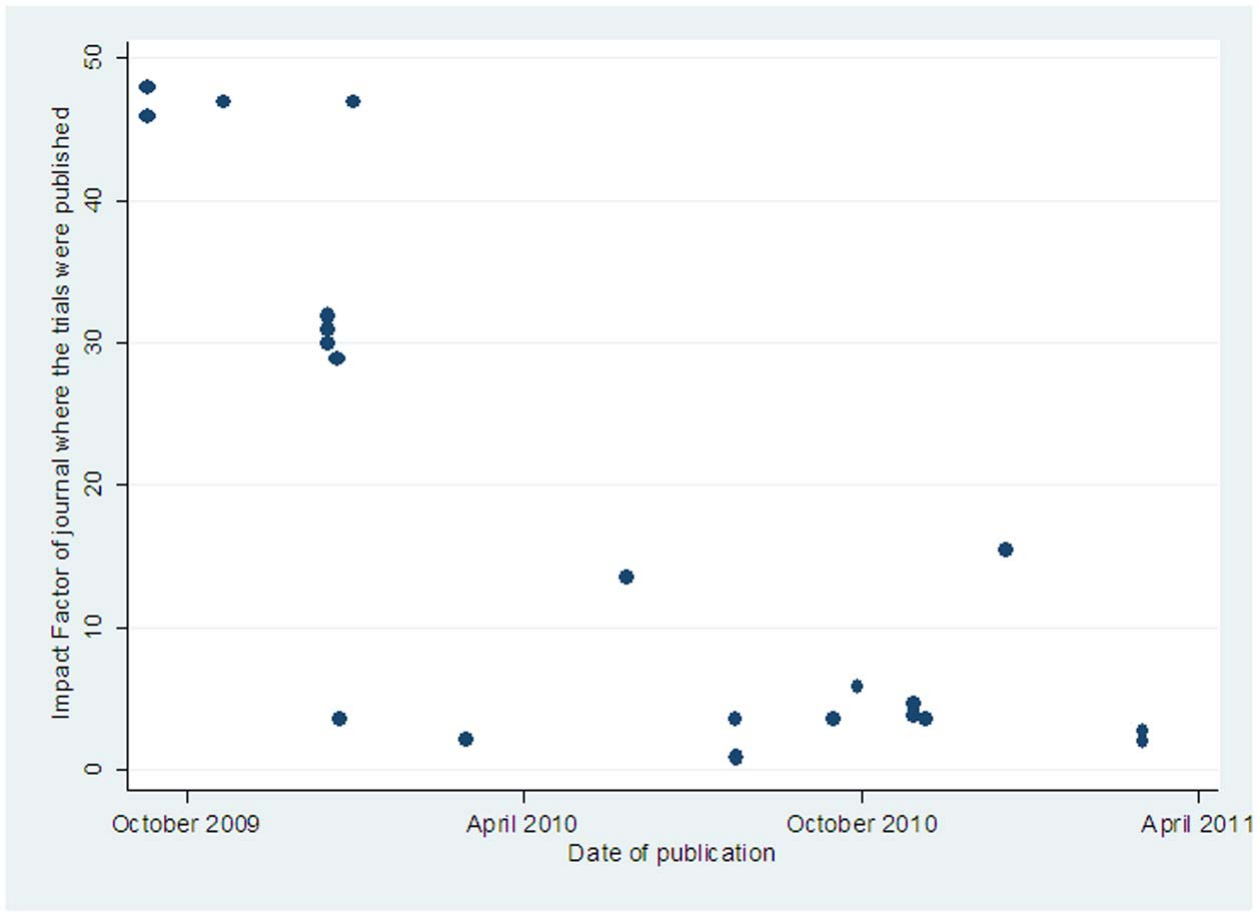
Scatter plot showing the impact factor of published randomized trials by time of publication.

As shown in Figure 4, the trials published later appeared in journals with lower impact factor (rank correlation coefficient −0.69, p<0.001). Eight of the 9 trials published in 2009 appeared in New England Journal of Medicine, Lancet, or JAMA. Only 2 of the 10 trials published in 2010 appeared in journals with impact factor above 6 and even these did not appear in any of the aforementioned 3 top-impact journals. The only two trials published to-date during 2011 were published together as a single paper (2 in 1) in a journal with impact factor less than 2.5.

## Discussion

Two years after the emergence of the influenza 2009 H1N1 pandemic and well after the end of both the 2009–2010 and 2010–2011 seasons only a minority of the registered randomized evidence on the potential vaccines has been published. The global response to the pandemic was ultrafast ^1^ and this included the early launch of numerous randomized trials for testing many different vaccine formulations. However, very limited randomized evidence was published in the peer-reviewed literature by the time major decisions were made in the fall of 2009 about the use of these vaccines [3]. Peer-reviewed data appeared in the highest-impact journals on over 15,000 participants by the end of 2009, but relatively limited evidence was published in 2010 or 2011 and none of the trials launched after October 2009 have been published as of June 2011, well after the 2010–2011 influenza season has finished. Trials were generally completed promptly, with a median time of 5 months from starting until completion. This is not surprising given the relatively simple design of these trials with short-term follow-up. The major problem was the delay after completion of the trials. Trials that started late and similarly trials completed late had limited chances of getting published.

Other investigators have described that for yet another major epidemic, SARS, the proportion of relevant research published during the epidemic was limited [4], [5]. Most literature on SARS was published after the epidemic had ceased to be a problem. It is unknown what portion of the conducted research was actually never published at all, as interest in SARS declined sharply in later years in most circles. However, the core literature of SARS did not involve randomized trials, while vaccine trials were of pivotal interest for the 2009 H1N1 pandemic.

The publication of clinical trial results is generally considered an ethical imperative. Much as a survey with 30–40% response rate is considered of questionable validity, a randomized trials agenda where only 38% of the data have been published poses concerns. Lack of publication of randomized trials, often coupled with a biased selection against trials with specific results, is well documented across very diverse fields [5]–[13]. Only 42% of an unselected sample of trials completed in 2005 had been published by the end of 2007 [14]. Randomized controlled trials, in particular phase III trials, can vary substantially on the time they take to conduct, analyze and publish. This time includes enrolment, patient follow-up, data analysis, manuscript preparation, peer-review, possible rejections, and publication phases [10]. For trials that require substantial follow-up, results may be published many years after the trial starts [10].

For vaccine trials where timely evidence is needed, the evaluation of the primary immunogenicity and short-term safety outcomes can be performed quickly and trials are completed in minimal time. Therefore the rate-limiting steps are manuscript preparation, review and publication of the results. Our data do not allow us to know with certainty which of these steps in the publication process may have been most retarding for influenza H1N1 vaccine trials. However, it is reasonable to suspect that authors, reviewers and journals may all show urgency in writing, reviewing and publishing results, if these become available early on. This is proven by the very rapid publication of the very first few trials, all of which were published in record time in the most prestigious medical journals and attracted enormous attention in 2009 [15]. The three trials published in New England Journal of Medicine in 2009 [16]–[18] received according to the Thompson Reuters Web of Knowledge 80, 58, and 58 citations, respectively, within the first year from their publication. However, this was just the tip of the iceberg of the randomized evidence on this topic. Interest in the other trials diminished and faded over time, in particular after the fall of 2009. Later published trials appeared in journals of far lesser citation impact. By 2011 two trials were published as a single paper in a low impact-factor journal, while trials of similar magnitude could have been published in a major journal in 2009.

Eventually, less than 30% of the trials registered in 2009–2010 were published by mid-2011. This lack of published data for the majority of the evidence creates difficulties in systematically appraising the overall randomized agenda of influenza H1N1 vaccines [19]. Moreover, numerous formulations have been developed from at least 14 different companies and it is not easy to extrapolate inferences from one formulation to another. Fragmentation and lack of publication shrink the evidence-base on a topic of major public health importance.

Some limitations should be acknowledged. First, we do not know the results of the unpublished trials and few of them (n = 5) seem not even completed yet. There is a substantial literature in other fields that unpublished or late-published trials have less favourable or even “negative” results as compared with more rapidly published trials [10], [12]. However, we have no evidence for such a bias in 2009 H1N1 vaccine trials. Most of these trials do not have results that can be categorized as “positive” and “negative” anyhow, since they compare different doses and formulations and, with the exception of very low doses, they are likely to generate substantial immunogenicity. Moreover, safety seems to have been very well established currently, at least in the short-term, based on observational studies of thousands of people who received 2009 H1N1 vaccines [20]. However, the lack of published information on the majority of the randomized data on immunogenicity does not allow estimating with high reliability the relative merits of different formulations.

Second, it is possible that some additional trials exist that are not registered. Then our reported non-publication rates may even underestimate the magnitude of this problem. For example, an updated search at the time of the revision of this manuscript (October 25, 2011) identified two otherwise eligible trials [21], [22] that were recently published (in August and September 2011, respectively) and that made no mention to registration. This is despite the fact that the publishing journals for these trials have instructions to the authors asking for registration of randomized trials and documentation of the registration number. The denominator of the total number of launched unregistered trials is by default unknown. Otherwise, the quality of the registry-recorded information is probably adequate. One potential exception is that, as we acknowledge in the Methods, information on the time of completion of a trial based on registry information can be sometimes tenuous, thus analyses using the date of completion require extra caution.

Finally, some additional trial results may have been made available by companies to select committees of key organizations and experts/insiders in the H1N1 field. Such insider-views and privileged communications are typical in almost any medical field. However, this does not negate the importance of publishing the results in the wider peer-reviewed literature. When we checked for such publicly available information, we found only scarce and fragmented data on H1N1 trial results at the FDA website, and only a minority of the trial reports posted on the EMA website reported vaccine compositions (covered under manufacturer's codes), thus it was impossible to ascertain which formulations are most immunogenic or safe [23]–[25]. Having widely accessible data in regulatory agencies and also in the peer-reviewed literature may diminish the publication delay issue. Such public data transparency will also help address concerns about the differences observed between regulatory-submitted and literature-published results that have been documented for medication trials [26]–[27].

Expedited posting, review and timely online publication of randomized results may also be feasible, employing evolving structures such as PLoS Currents: Influenza [28]. However, one has to ensure that such online options employ also rigorous and transparent peer-review and also are utilized for this purpose. To our knowledge, none of these trials were posted in PLoS Currents: Influenza. A perusal of the 75 articles in PLoS Currents: Influenza as of October 24, 2011 shows that none of them are randomized clinical trials on humans (there is only one trial on pigs). Investigators may feel that there is an opportunity cost in writing up manuscripts for publication if they feel that they would no longer be attractive and cited and would most likely be published only in low impact journals. However, one has to find incentives for the majority of trials to become published after peer-review, including the majority of trials that did not make it into publication during the early phase of golden opportunity for publication in major journals. This information may be of critical importance in giving a more comprehensive picture of the available evidence for the future, for any subsequent pandemics by the same virus. Remedying the publication system for such trials would also be critical for improving the completeness of the randomized evidence for future pandemics by other infectious agents.

### Ethics

The study did not require ethics approval.

### Data sharing

Technical appendix with all the data on the 73 registered randomized controlled trials on 2009 A(H1N1) influenza vaccines available from the corresponding author.
